# Evaluation of the early warning alert and response system mobile pilot for East and Central Darfur, Sudan

**DOI:** 10.11604/pamj.2026.53.99.49910

**Published:** 2026-02-25

**Authors:** Kazuki Shimizu, Muhammad Ali Raja, Muntasir Mohammed Osman, Elfadil Mohammed Mahmoud, Mazza Abasher Alzain, Rizwan Ayub, Marcel Woung, Sara Ahmed, Egmond Samir Evers, Sherein Elnossery, Douaa Fouad Osman Ibragem, Siddeg Khalafalla Ahmed Mustafa, Liesbeth Aelbrecht, Muhammad Fawad Khan, Simon Kaddu Ssentamu, Hala Khudari, Hani Haidar, Shible Sahbani, Haitham Mohamed Ibrahim Awadallah, Boris Igor Pavlin

**Affiliations:** 1 World Health Organization Country Office in Sudan, Port Sudan, Sudan,; 2 World Health Organization Regional Office for the Eastern Mediterranean, Cairo, Egypt,; 3 World Health Organization Health Emergencies Programme, Geneva, Switzerland,; 4 Federal Ministry of Health, Port Sudan, Sudan

**Keywords:** Sudan, Darfur, conflict, cholera, outbreak, surveillance

## Abstract

In conflict-affected areas like Darfur in Sudan, the disruption of health infrastructure and disease surveillance systems has severely hindered timely detection and response to disease outbreaks. To address the gap, the Sudan Federal Ministry of Health (FMoH), with support from the World Health Organization (WHO) and Health Cluster partners, piloted an emergency disease surveillance system using WHO´s Early Warning Alert and Response System Mobile Application (EWARS Mobile) in East and Central Darfur from mid-2024. The pilot phase was evaluated using standard WHO and the United States Centers for Disease Control frameworks for surveillance system assessment, focusing on quantitative epidemiological attributes and qualitative stakeholder feedback. Data from health facilities managed by partners were analyzed, and stakeholders´ perspectives were gathered through remote consultations and two evaluation meetings in December 2024 and February 2025. Over the pilot period, 52934 cases of ten monitored syndromes were reported from a total of 166663 consultations. The system demonstrated high completeness and low but gradually improving timeliness, with enhanced geographic representativeness and the generation of actionable alerts, enabling communication and data sharing through seamless integration with the national surveillance platform. Though several epidemiological attributes, such as sensitivity and specificity, could not be fully assessed, the EWARS Mobile was found to be highly useful, acceptable, flexible, and simple, with minimal resource requirements and strong partner interest in expansion. The EWARS Mobile pilot effectively generated critical health intelligence in East and Central Darfur States, hard-to-reach areas in Sudan, underscoring the system´s feasibility and sustainability in low-resource, volatile settings where no functional surveillance mechanisms existed following the escalation of the conflict.

## Introduction

In fragile, conflict, and violence-affected settings, the displacement of populations, the collapse of health systems, and the destruction of health infrastructure, including targeted attacks on healthcare facilities, significantly increase the risk of acute public health events, particularly outbreaks of infectious diseases. In such contexts, timely detection, verification, risk assessment, and response are critical to preventing the escalation of health threats and mitigating widespread morbidity and mortality [1]. Early Warning Alert and Response Systems (EWARS) are essential components of public health intelligence, enabling rapid identification and containment of outbreaks [1,2]. Their importance is amplified in settings where conventional surveillance mechanisms are disrupted, compromised, or entirely non-functional [1].

**Sudan: continuous pressure on the health system:** since April 2023, the conflict in Sudan has disrupted medical and public health services and damaged the health information systems infrastructure [3,4]. Particularly in hard-to-reach states in the Darfur and Kordofan regions, there was an absence of any reliable alternative data source for these states for over a year, creating a blind spot for disease outbreaks [2,3]. Reports of suspected measles, cholera, and hepatitis E among refugees and local populations in non-reporting areas [2,5-7] highlighted the urgent need for a lighter, more adaptable disease surveillance system.

**The EWARS Mobile implementation in Sudan:** in response, the World Health Organization (WHO), with support from Health Cluster partners and the Sudan Federal Ministry of Health (FMoH), piloted an emergency surveillance approach using WHO´s EWARS Mobile Application (EWARS Mob App) in East and Central Darfur from mid-2024 to assess its feasibility for broader implementation [2,3,8].

Early Warning Alert and Response Systems (EWARS) Mobile pilot for East and Central Darfur included immediate and weekly reporting [2]. Under the indicator-based surveillance (IBS), five conditions were categorized for immediate notification, and six conditions were subject to weekly reporting (Table 1). Under event-based surveillance (EBS), any other unusual public health events were set to be immediately notified. Assigned health workers recorded cases daily for immediately notifiable conditions and weekly for surveillance of key conditions without interrupting clinical care, based on the epidemiological week, starting on Saturday and ending on Friday [2]. Immediate alerts and weekly summaries were submitted to a central system, integrated via application programming interface (API) into Sudan EWARS, the national system launched in August 2023 for 10 accessible states [9]. Signals were reviewed by the FMoH and WHO, with partners involved in verification and response as needed.

**Table 1:** syndromes monitored by the Early Warning Alert and Response System Mobile Application (EWARS) Mobile pilot in East and Central Darfur, 31^st^ August 2024 – 24^th^ January 2025

Reporting frequency	Syndrome	Number of cases	Frequency (cases per 100 cases of all syndromes)
Immediate reporting	Acute flaccid paralysis	11	0.02
	Suspected measles	88	0.17
	Suspected cholera	3	0.01
	Suspected mpox	9	0.02
Weekly reporting	Suspected meningitis	0	0.00
	Bloody diarrhea	3403	6.4
	Acute jaundice syndrome	79	0.15
	Severe acute respiratory infection	15679	29.6
	Suspected malaria	7000	13.2
	Confirmed malaria	26662	50.4

Analyzed data were disseminated to partners and the Federal Ministry of Health (FMoH) through weekly bulletins [2,8]. Bi-weekly coordination meetings involving partners, FMoH, and WHO, specifically focused on EWARS Mobile for Darfur, were held to review and discuss the evolving epidemiological situation [2,8]. Key findings were also shared and discussed during the bi-weekly Darfur sub-national health cluster meetings.

To inform the expansion of the system across all Darfur States, the performance of the EWARS Mobile pilot in East and Central Darfur was initially evaluated in December 2024, followed by a follow-up in February 2025. Stakeholders convened for a dedicated evaluation meeting to review the pilot phase, during which key findings, challenges, and opportunities were discussed. While the actual implementation process has been documented elsewhere [2], the details of the evaluation methodology and its results have not yet been published. This report presents a comprehensive overview of the evaluation approach, key findings, and insights gained from the pilot implementation.

## Methods

**Setting:** the population under surveillance comprised individuals seeking care at health facilities supported by trained reporting partners and incorporated into the EWARS system in East and Central Darfur States.

**Evaluation approach:** by learning from precedent examples in different contexts [10-13], the evaluation for the EWARS Mobile pilot surveillance system for East and Central Darfur was conducted based on standard frameworks for the evaluation of communicable diseases by the WHO and the United States Centers for Disease Control and Prevention [14,15] and covered quantitative epidemiological attributes and qualitative stakeholder feedback. The period from 31^st^ August 2024 to 24^th^ January 2025 was reviewed. The evaluation was undertaken through a remote review of EWARS Mobile records and through users' opinions, with stakeholders involved in the system implementation. Most attributes were evaluated directly by examining the system's data, with inputs provided based on reporters' user experiences before and through the evaluation meetings regarding two questions: (i) whether you have found the system easy to use or not; (ii) whether you recommend any changes to the system. The quantitative data were analyzed using Microsoft Excel 2021 (Microsoft Coop. Redding. USA). Qualitative data were analyzed based on manual thematic analysis, and findings were summarized based on thematic areas.

**Epidemiological attributes:** system attributes were evaluated under FMoH´s guidance and with suggestions. The evaluation focused on standard epidemiological attributes: completeness, timeliness, representativeness, sensitivity, specificity, positive predictive value, usefulness, acceptability, flexibility, and simplicity, which were defined as follows, by referring to the global standard frameworks [14,15].

Completeness: proportion of registered partners and partners´ facilities submitting weekly reports, by measuring from the time of their first report after being trained, and not counting the weeks before being trained; timeliness: proportion of weekly reports submitted within three days after the end of the epidemiological week in Sudan (i.e., Monday night); representativeness: to what extent the system accurately captures and describes disease occurrence over time and its distribution across the population; sensitivity: ability to detect true cases and outbreaks and monitor changes over time; specificity: ability to avoid false positives; positive predictive value: proportion of reported cases that are confirmed true cases; usefulness: system´s capacity to provide useful information and inform action; acceptability: users (i.e., reporters´ and partners´) satisfaction and willingness to participate in the system; flexibility: ability to adapt to modifications (e.g., adding new conditions, revising case definitions, changing reporting indicators and locations, and integrating with other systems with minimal resources); simplicity: ease of system use, including reporting burden and operational stability, while meeting surveillance objectives.

**Ethical considerations:** the evaluation was conducted with approval from the Federal Ministry of Health, Sudan. The system did not collect any identifiable patient information; all data were anonymized and encoded, eliminating any risk of identifying participants.

## Results

Between 31^st^ August 2024 and 24^th^ January 2025 (between epidemiological week 36, 2024 and week 3, 2025 in Sudan), 52934 cases of the ten monitored syndromes were reported following 166663 consultations from 148 health facilities (Table 1). Among monitored syndromes, confirmed malaria was the most common reported condition (26662 cases, 16.0% of consultations), followed by severe acute respiratory infection (SARI) (15679 cases, 9.4%), suspected malaria (7000 cases, 4.2%), and bloody diarrhea (3403 cases, 2.0%) (Table 1). Key findings of epidemiological attributes are summarized below.

**Completeness:** as of December 2024, five out of 17 known health partners in Central and East Darfur participated in the EWARS Mobile pilot. Overall, there was great heterogeneity in completeness across different reporting partners, with two partners providing a large number of reports with completeness of 81% and 47%. Other partners had completeness rates below 10%, including two partners that dropped out shortly after their initial submission. Dropping these partners increased completeness to 38%. Overall, completeness amongst participating partners was assessed as moderate, given the short duration of the pilot and the extremely challenging accessibility and connectivity.

Through the first three weeks in January 2025, following FMoH´s recommendations to intensively engage with partners to increase reporting, and through efforts primarily by WHO for partner engagement, with the support of FMoH, two additional partners were identified, and the total number of known partners in Central and East Darfur has reached 19. Among these, 17 were engaged, with 16 reported at least once. More than 270 reports were submitted from partners within the first three weeks in 2025 (Sudan epidemiological weeks 1-3, 2025), exceeding the total amount of reports through September - November 2024. The facility-level completeness increased to 59%, with each partner´s performance summarized ranging from 33% to 100% (Table 2).

**Table 2:** completeness of Early Warning Alert and Response System Mobile Application (EWARS) Mobile reporting in Central and East Darfur, Sudan, January 2025

Partner	Registered sites	Reports submitted	Expected reports	Completeness
Partner A	3	4	9	44%
Partner B	12	12	36	33%
Partner C	15	34	45	76%
Partner D	5	15	15	100%
Partner E	11	18	33	55%
Partner F	1	2	2	100%
Partner G	11	13	33	39%
Partner H	2	6	6	100%
Partner I	9	14	27	52%
Partner J	14	15	42	36%
Partner K	6	14	18	78%
Partner L	8	13	24	54%
Partner M	7	21	21	100%
Partner N	18	30	54	56%
Partner O	1	3	3	100%
Partner P	20	40	60	67%
Total	143	254	428	59%

**Timeliness:** it was low throughout the pilot period, with 27% in September - November 2024. In January 2025, there was a fluctuation or the timeliness, with 33% maximum in the Sudan epidemiological week 2 (Table 3).

**Table 3:** timeliness of Early Warning Alert and Response System Mobile Application (EWARS) Mobile Reporting in Central and East Darfur, Sudan, January 2025

Week	Reports on time (%)	Late reports (%)	Very late reports (%)	Total reports
Week 1	2 (2%)	35 (36%)	61 (62%)	98
Week 2	28 (33%)	23 (27%)	34 (40%)	85
Week 3	21 (24%)	50 (56%)	18 (20%)	89

**Figure 1:**
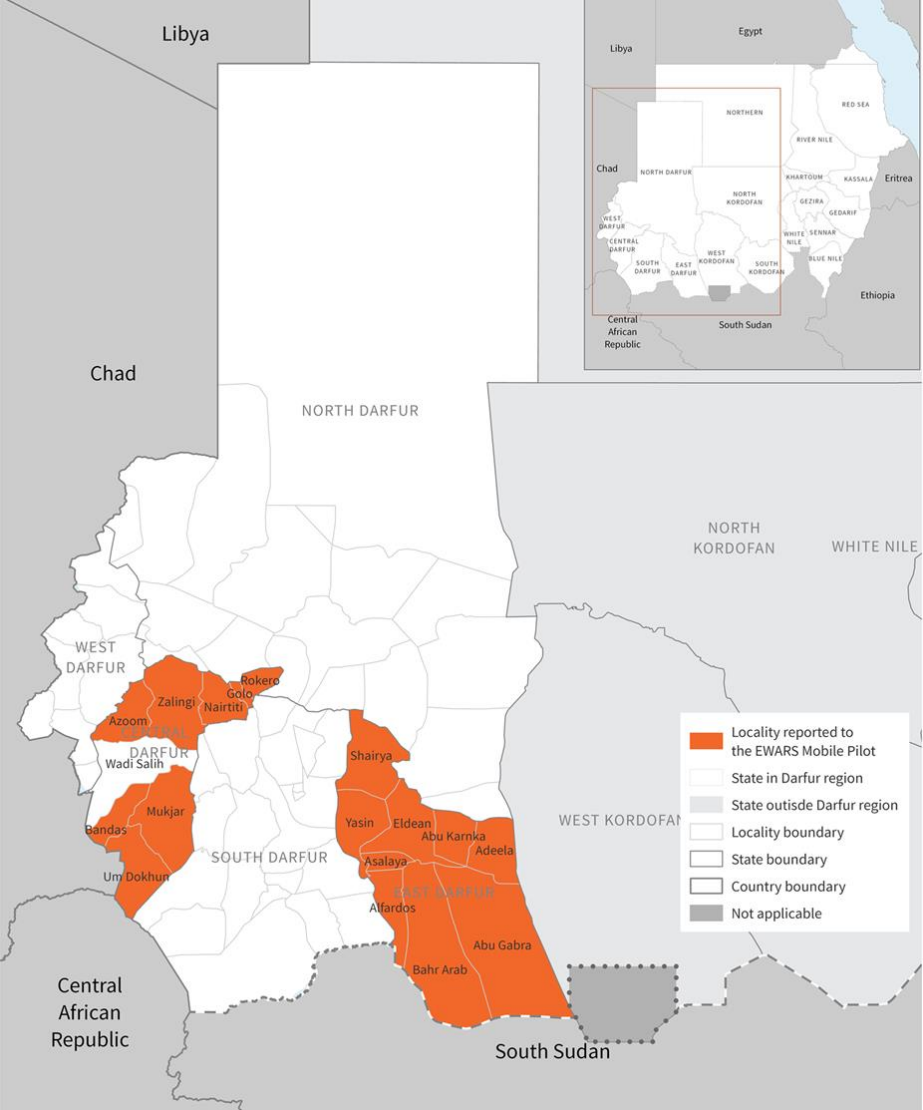
localities reported to the Early Warning Alert and Response System Mobile Application (EWARS) Mobile Pilot in East and Central Darfur States, Sudan, September 2024 - January 2025

**Representativeness:** in September - November 2024, in East Darfur, six out of seven (86%) localities submitted at least one weekly report; in Central Darfur, this figure was 44% (four out of nine localities). In January 2025, the geographic representativeness improved dramatically, with all nine localities (100%) in East Darfur and eight out of nine localities (88%) in Central Darfur submitting at least one weekly report (Figure 1).

**Sensitivity:** it could not be fully evaluated due to a lack of access to facility registers and the impossibility of comparing reports with them. However, the system successfully captured several alerts, such as acute flaccid paralysis and suspected measles, which were consistent with previously reported and confirmed cases, validated by the focal points from the Expanded Program on Immunization. No alerts for unusual events were raised, and no interviews with community informants were conducted to assess whether any such events occurred but failed to be reported.

**Specificity:** some initial indicator-based weekly reports were false due to a misunderstanding of case definitions. For example, many mild acute respiratory infection cases were reported as SARI. While specificity improved over time due to regular feedback to users to encourage strict adherence to case definitions, it was not possible to quantify the indicator-based specificity. With regard to alert specificity, several false alerts were triggered early in the pilot, especially for common diseases with high variability between facilities, such as malaria and bloody diarrhea, for which the specificity of alerts was subsequently increased through threshold adjustments. Some alerts were due to increases in total consultations rather than disease incidence, prompting the adoption of proportionate morbidity thresholds. As no event-based surveillance alerts were raised, it was not possible to assess whether any of these were false alerts. Overall, specificity improved over time but remained a challenge.

**Positive predictive value:** due to the lack of data on the sensitivity and specificity of individual diseases in the system, coupled with the lack of baseline prevalence data, positive predictive value could not be assessed.

**Usefulness:** the system was considered to have high usefulness. EWARS Mobile filled a critical data gap in East and Central Darfur, providing routine health intelligence and enabling early alerts for several conditions. It also helped confirm the absence of suspected cholera and suspected mpox between September and December 2024.

The platform facilitated communication among partners via WhatsApp and email, which were leveraged to discuss signals of outbreaks and provide rapid follow-up information on submitted reports. Data were integrated with the national EWARS system through an API, allowing data to be analyzed along with accessible states´ data in FMoH. The system also generated automatic weekly bulletins that were shared back to partners to provide an evidence base for decision-makers for their operational decision-making. User feedback highlighted the value of the system in tracking disease trends, enhancing coordination, and improving accountability towards FMoH, states´ ministries of health, and the affected populations, with suggestions for improving data quality.

**Acceptability:** in addition, high acceptability was evident through partner interest in expansion, willingness to use the system without incentives, and appreciation of the light reporting burden. At the beginning of the pilot implementation, in-person training was contemplated across the border in Chad to achieve high technical proficiency and partner buy-in; however, this option was determined to be logistically and cost-prohibitive. Although resorting to remote engagement hampered the initial process, it was eventually overcome through persistent bilateral online follow-up.

**Flexibility:** EWARS Mobile demonstrated very high flexibility. The system was operational with a minimal basic orientation for set-up and made it possible for anyone with the appropriate permissions to set up custom indicators, locations, reporting forms, and standardized reports without a background in information technology. The system allowed quick revision of disease lists (e.g., addition of mpox), integration of new partners and sites, and offline reporting from Android devices. Technical glitches, such as misalignment of reporting dates and duplication of names, had a minimal impact on operations and were rapidly identified and easily corrected. Offline functionality and minimal technical requirements made it suitable for the low-connectivity settings. Minor issues, such as confusion with login, were resolved quickly.

While facility-based reporting primarily focused on those who had access to health facilities, the system also accommodated EBS through the “Unusual Events” category, where any events not presented in the IBS list could be captured. This inclusion allowed for potential community-level coverage, although no community events were reported during the pilot.

**Simplicity:** simplicity was a key design feature, which was considered very high overall. Compared to the national platform with 28 disease categories and 265 weekly fields to enter, EWARS Mobile has 11 categories and 14 weekly fields. Users found the system straightforward and intuitive, given that it did not require validation before data were available to authorized users. The system supported offline entry, automated alerts, bulletins, and user-friendly interfaces. Common issues, such as login challenges, were addressed through peer support and group communication over WhatsApp (Meta, Inc., Menlo Park, USA), including troubleshooting sessions. Simplicity also contributed to sustainability, with no need for financial incentives or phone top-up credits, thus achieving cost savings. Targeted support and refresher training were suggested to improve partner uptake and retention.

## Discussion

The EWARS Mobile pilot in Central and East Darfur successfully generated critical health intelligence from areas previously lacking surveillance capacity. Despite operating with minimal resources, the system demonstrated feasibility and sustainability. Feedback from the Sudan FMoH in December 2024 highlighted several strengths. The system was commended for its simplicity, user-friendly interface, and offline functionality, which enabled operations in regions with limited or no network coverage. These features were particularly valued for facilitating understanding of the health status in hard-to-reach areas.

However, areas for improvement also existed to expand the system to additional states. Challenges were particularly noted in partner engagement, with concerns about limited commitment from partners. Recommendations included enhancing collaboration with partners, identifying and addressing underlying factors contributing to low reporting rates, and improving data quality, especially in terms of timeliness and detailed information sharing for immediately notifiable conditions. To strengthen sensitivity and specificity, refinement of case definitions, adjustment of alert thresholds, particularly for malaria using proportionate morbidity rather than historical averages, and targeted, structured, and comprehensive refresher training were suggested. The importance of involving State Ministries of Health in monitoring, evaluating alerts, and coordinating responses was emphasized, alongside the need for expanded data quality training and broader dissemination of the EWARS Bulletin to enhance usefulness and acceptability. No major concerns were raised regarding flexibility, and maintaining system simplicity was deemed essential to support partners with slower uptake and ensure continued capacity-building, including training.

Progress was evident in January 2025, marked by intensified outreach and engagement efforts by WHO and FMoH [2]. These included bilateral follow-up meetings to broaden partner involvement, the establishment of partner-specific WhatsApp communication channels, and rapid troubleshooting support primarily led by WHO [2]. These initiatives contributed to a notable increase in report submissions and the timely revision of the health facility list. A follow-up evaluation in February 2025 confirmed improvements in timeliness and representativeness, reflecting the positive impact of targeted interventions.

Given the system´s growing utility, minimal resource requirements, and the absence of viable alternatives, continued implementation of EWARS Mobile in the pilot areas was strongly recommended. In addition, the system expansion to the remaining Darfur states, accompanied by targeted technical enhancements and regular, comprehensive reviews through fortnightly coordination meetings involving FMoH, partners, and WHO, was agreed. Since the pilot, notable improvements have included strengthened partner engagement, technical upgrades, and an updated list of notifiable diseases (i.e., addition of suspected viral hemorrhagic fever and diphtheria) in response to emerging public health threats within Sudan and neighboring countries.

**Limitations:** the EWARS Mobile pilot faced several operational and contextual challenges that constrained its full evaluation and implementation. Due to insecurity, on-site evaluations were not feasible, limiting the ability to assess the system´s performance comprehensively. As a result, key surveillance attributes such as sensitivity, specificity, and positive predictive value could not be adequately evaluated. Improving sensitivity and specificity requires systematic evaluations, ideally conducted through on-site reviews, as has been done in the past [16]. Additionally, the linkage between EWARS Mobile and the revitalization of rapid response teams remained underdeveloped, which hampered timely verification and risk assessment. The evaluation of actual response activities to acute public health events was also beyond the scope of the pilot.

As highlighted in the previous study [17], the underutilization of EBS emerged as a notable gap. Although EBS was designed to capture signals such as clusters of unusual illnesses, unexplained deaths, and environmental hazards, with the potential to cause harm to human health, or any suspicion, or diagnosis of any other concerning communicable diseases that did not meet IBS criteria, including those captured as rumors from the community, no community-level events were reported during the pilot, highlighting the need to strengthen representativeness beyond facility-based reporting, which primarily reflects populations with access to health services. Reporting completeness was further affected by limited partner participation, where 12 partners did not respond to initial engagement efforts, and one partner ceased operations shortly after training. Timeliness was impacted by weekend clinic closures and delayed mobile network access [2]. Another likely factor might be the design of the system in Sudan, where the reporting was through an intermediary at a higher level, such as a surveillance or monitoring officer, who first had to collate reports from the field, not primarily done directly by frontline clinic staff.

## Conclusion

The EWARS Mobile pilot successfully generated critical public health data from Central and East Darfur, where previously there was a lack of reliable surveillance due to conflict and inaccessibility. The system proved functional with minimal resources. While areas for improvement were identified in December 2024, especially the need to engage more partners, no insurmountable obstacles to implementation were found. Through January 2025, advocacy efforts were highly effective and yielded excellent results, with sustained interest from partners in expanding the system to additional locations. Following the pilot evaluation, it was agreed that the EWARS Mobile system underwent several enhancements, including further improved partner engagement, technical upgrades, and an updated list of notifiable diseases to address emerging threats within Sudan and neighboring countries. Given its demonstrated effectiveness in hard-to-reach settings with low resources, and the lack of viable alternatives, the FMoH fully endorsed the system´s expansion across all-Darfur States, with continued support from WHO and partners.
